# Exploring the knowledge, perception, and practice of community pharmacists in Saudi Arabia toward pharmacovigilance and adverse drug reaction reporting. A nationwide survey

**DOI:** 10.1038/s41598-024-55664-8

**Published:** 2024-02-27

**Authors:** Amani Khardali

**Affiliations:** 1 Department of Clinical Pharmacy, College of Pharmacy, Jazan University, 45142 Jizan, Jazan Saudi Arabia; 2 Present Address: Pharmacy Practice Research Unit, College of Pharmacy, Jazan University, 45142 Jizan, Jazan Saudi Arabia

**Keywords:** Adverse drug reaction reporting, Community pharmacists, Pharmacovigilance, Saudi Arabia, Environmental social sciences, Health care, Health occupations, Medical research, Risk factors, Signs and symptoms

## Abstract

One of the significant worldwide health problems associated with pharmacovigilance is the under-reporting of adverse drug reactions (ADRs). Reporting suspected ADRs is essential to ensure patient safety, medicine safety, and healthcare quality. The new policy in Saudi Arabia emphasizes pharmacists taking a new clinical role, which may facilitate and improve the documentation of ADRs. Therefore, this study aimed to assess the knowledge and perception of community pharmacists towards the ADRs and their reporting practice in Saudi Arabia. A cross-sectional study using a structured self-administered questionnaire was administered to community pharmacists working in Saudi Arabia. Data were analyzed using descriptive and inferential statistics to identify the association between perceptions and ADR reporting practices. A *P* value < of 0.05 was considered statistically significant. A response rate of 43% (n = 163) was achieved, of whom 55.2% demonstrated knowledge of PV. Only 16% of community pharmacists were aware of the responsible center for monitoring and collecting ADRs in Saudi Arabia. The key facilitator was offering incentives to pharmacists, and the lack of time was found to be a key barrier among reporter community pharmacists. Positive attitudes toward pharmacovigilance and ADR reporting were expressed by community pharmacists. The findings of this study emphasize the further need for education and training programs and simplifying the ADR reporting process used in Saudi Arabia to enhance the reporting practice.

## Introduction

Adverse drug reactions (ADRs) significantly impact the healthcare systems in terms of morbidity, mortality, and financial burden that might be associated with treating patients with ADRs^1,2^. According to World Health Organization (WHO), an ADR is defined as "any response to a drug which is noxious and unintended, and which occurs at doses normally used in man for prophylaxis, diagnosis, or therapy of disease, or the modification of physiological function"^3^. The monitoring of ADRs is considered the fundamental concept of pharmacovigilance, which is defined as the "pharmaceutical science and activities relating to the detection, assessment, understanding, and prevention of adverse events or any other drug-related problems"^3^. Pharmacovigilance is a continuous process that is usually started from the initial stages of the drug development process to clinical trials and post-marketing surveillance activities. In 2009, Saudi Arabia established the National Pharmacovigilance Center (NPC) under the umbrella of the Saudi Food and Drug Administration (SFDA), which later became a full member of the WHO's Uppsala Monitoring Center^4^.

The NPC-SFDA is responsible for collecting the spontaneous reporting forms via the Saudi Vigilance System (SVS). The system's primary goal is to report and monitor the safety and effectiveness of chemical and biological medications, medical devices, food and herbal supplements, and cosmetics products. The SFDA accepted the spontaneous reporting of post-marketed medications from healthcare professionals, manufacturers, and patients^5^. Meanwhile, the NPC announced and launched several initiatives, including reaching 30,000 ADR reports in 2018^4^; however, a recent study reported that 17,736 reports were submitted to SFDA in 2017^6^. Furthermore, a previous study revealed that there is still an underreporting of ADRs by HCPs; for example, 10.2% of community pharmacists in Saudi Arabia admitted to submitting an ADR report to NPC^7^. The author of this study has stated that the reporting of ADRs is highly related to the knowledge and attitude of healthcare professionals (HCPs) (i.e., pharmacists)^7^.

Community pharmacy is one of the pharmacists' most extensive work sectors in Saudi Arabia^8^. Pharmacists employed in the community sector are primarily responsible for dispensing and counseling services for prescription and over-the-counter (OTC) medications^8^. In 2016, the Saudi Ministry of Health (MOH) presented over 40 healthcare initiatives as part of the Saudi Vision 2030^9^. Of these 40 initiatives, 15 were related to advancing pharmacy practice and pharmaceutical care services. As a result, the Pharmacy practice has evolved, with pharmacists becoming more patient-centered and clinically focused approaches than a product-centered approach^9^. This could offer diverse opportunities for pharmacists in Saudi Arabia to extend their routine practice beyond dispensing and preparing medications and into ensuring the safe and effective use of medicines through the provision of patient counseling, ADR reporting, and drug therapy monitoring. Indeed, among the HCPs, community pharmacists are the most accessible to the public regarding medication information and safety Fields^10^. They must have essential knowledge, attitudes, and practice toward medication safety and reporting ADRs^11^. Hence, this study aimed to understand and explore community pharmacists' knowledge and perspectives on ADR reporting and their reporting practices in Saudi Arabia, specifically focusing on the possible barriers and facilitators to reporting.

## Method

Experience, knowledge, and perceptions of community pharmacists working in Saudi Arabia toward reporting ADRs were assessed by using a cross-sectional online survey.

The questionnaire was adopted and developed from an existing questionnaire that was used in a previous study that assessed perceptions of community pharmacists in Australia towards reporting ADRs^12^. This questionnaire was developed to include three main aspects linked to the aim of this study: knowledge of pharmacovigilance, perceptions towards ADR reporting, and current practices of ADR reporting. However, some of the questions in this questionnaire were rephrased to fit the regulations and practices within Saudi Arabia (Supplementary Material). The final version of the questionnaire was reviewed and validated by a subset of community pharmacists (n = 3) and pharmacy academics (n = 5), as well as the internal consistency of the questionnaire was assessed using Cronbach's alpha, and the result was approximately 0.7 indicating good reliability of the research questionnaire.

The self-administered questionnaire contained four sections; the demographic information section consisted of 5 questions. The knowledge section involved ten multiple-choice questions with one correct answer. The perceptions section included 11 Likert item questions (5-point items). Finally, the practice-related questions had three multiple-choice questions and one free-text question.

According to the statistical data conducted by the Ministry of Health in 2021, there were 17,815 pharmacists working in community pharmacies in Saudi Arabia^13^. The sample size was then determined by using a Raosoft sample size calculator^14^, with a confidence interval of 95% and a margin of error equal to 5%. The estimated sample size of 377 pharmacists was found to be required for this study.

Community pharmacists were invited to participate through social media posts (i.e., Community pharmacy Telegram groups) and by requesting two big chain community pharmacies to disseminate the questionnaire to their employees. Participation was voluntary, and the participant information sheet was made available to all the respondents. A question was added at the beginning of the questionnaire, asking if they were interested in participating and completing it. The return of the questionnaire was considered as implied consent. No reminders were allowed to be sent to the community pharmacists because of the internal policies of the chain pharmacies, which negatively impacted the total number of respondents.

Statistical analyses of the collected data were performed using IBM SPSS (version 25.0), and descriptive analysis (frequencies and percentages) was performed for all variables included in the study. The competitive analysis using the chi-square test was employed to compare the difference in the perceptions between pharmacists who have either observed or reported ADR and those who have not observed or reported any ADR, and significance was considered if the *p*-value was less than 0.05 (5% *p*-value). In addition, content analysis was used to analyze the qualitative data provided in the free-text questions.

### Ethics approval

Although this is not a clinical trial involving individual participants, this study was conducted in compliance with Ethical Standards**.** Ethical approval was granted from the Standing Committee for Scientific Research at Jazan University, Saudi Arabia (REC-43/11/272), and informed consent was obtained from all respondents.

## Result

A total of 163 community pharmacists responded and returned the questionnaire with a response rate of 43%. The majority of respondents were male pharmacists,147 (90.2%); other key demographic data are explained in Table 1. There was no significant correlation between age and reporting ADRs (rs = −0.125, *P* = 0.08); nevertheless, a positive significant correlation was found between years of experience and ADRs reporting (rs =  + 0.212, *P* = 0.04).

**Table 1:** Respondent Demographics (N = 163).

Demographics		N%/m ± SD
Gender	Male	147 (90.2%)
	Female	16 (9.8%)
Age		31 Years ± 3.8 (R: 24–45)
Nationality	Saudi	35 (21.5)
	Non-Saudi	128 (78.5)
Qualification	B. Pharm	131 (80.4%)
	PharmD	31 (19%)
	Postgraduate	1 (0.6%)
Year of experience		7 years ± 3.9 (R: 1–21)

### Knowledge about PV and ADR reporting systems in Saudi Arabia

Most respondents (n = 90, 55.2%) were able to define the PV correctly. When asked about the examples of ADRs, 87 (53.4%) selected the correct examples of adverse events. Although most of the respondents, 96 (58.9%), knew the type of ADRs that SFDA wanted them to report, only 26 (16%) of them were able to identify the system at the SFDA that is responsible for the ADRs report collection and analysis. The majority of respondents, 118 (72.4%), were known who had the right to send the ADRs report to SFDA, and 89 (54.6) of the respondents were aware of how the SFDA communicates important drug safety-related information with HCPs and the general public. In addition, 83 (50.9%) of the respondents were able to identify the correct phase of a clinical trial where the majority of ADRs related information is collected.

However, a few of the respondents, 17 (10.4%), were able to differentiate between Adverse events and ADRs, and 16 (9.8%) of them were able to identify the serious adverse event that needed to be reported to SFDA. As well, only 32 (19.6%) of respondents correctly identified what SFDA considered as common safety issues involving the drug in the market and need documentation.

### Perceptions towards PV and ADRs reporting

The majority of the respondents, 123 (75.5%), believed that reporting the ADRs is essential for patient care; 52 (31.9%) community pharmacists agreed that ADR reporting consider a professional obligation. A total of 107 (65.6%) community pharmacists agreed that PV should be taught in Pharmacy schools in Saudi Arabia. A total of 105 (63.2%) respondents were willing to report ADRs if they received rewards. Out of 163, 107 pharmacists mentioned that the lack of time in their practice represents a key barrier to reporting ADRs. Table 2 shows the details of the perceptions towards PV and ADRs reporting.

**Table 2:** Perceptions toward Pharmacovigilance and Reporting the ADRs.

Questions	Strongly agree	Agree	Neutral	Disagree	Strongly disagree
Reporting ADRs is important for patient care	43 (26.4%)	80 (49.1%)	7 (4.3%)	2 (1.2%)	31 (19%)
Reporting ADRs should be mandatory for community pharmacists	34 (20.9%)	18 (11%)	46 (28.2%)	34 (20.9%)	31(19%)
Pharmacovigilance should be taught in undergraduate pharmacy programs at universities	59 (36.2%)	48 (29.4%)	27(16.6%)	9 (5.5%)	20 (12.3%)
Professional bodies (e.g., the Saudi pharmaceutical society) should organize workshops or training sessions to cover the importance of ADR reporting	71 (43.6%)	38 (23.3%)	32 (19.6%)	3 (1.8%)	19 (11.7%)
I have a professional obligation to report ADRs	35 (21.5%)	17 (10.4%)	55 (33.7%)	36 (22.1%)	20 (12.3%)
I currently have sufficient knowledge and training on how to report ADRs	46 (28.2%)	15 (9.2%)	57(35%)	20 (12.3%)	25 (15.3%)
I would be encouraged to report more ADRs if it was: Rewarded	58 (35.6%)	45 (27.6%)	39 (23.9%)	4 (2.5%)	17 (10.4%)
General education is needed on the importance of pharmacovigilance	62 (385)	26 (16%)	52 (31.9%)	6 (3.7%)	17 (10.4%)
I do not have the time to report ADRs as part of my professional practice	59 (36.25%)	48 (29.4%)	28 (17.2%)	5 (3.1%)	23 (14.1%)
I fear that there may be legal consequences if I report an ADR to the SFDA	65 (39.9%)	28 (17.2%)	48 (29.4%)	9 (5.5%)	13 (8%)
There are no results or actions taken based on the ADRs that I report	62 (38%)	26 (38%)	52 (31.9%)	6 (3.7%)	17 (10.4%)

### Current practice

The majority of the respondents, 76 (46.6%) vs. 77 (47.2%), had observed and reported ADRs in their current practice to the SFDA at least once per month, respectively. Table 3 shows the details of community pharmacists' practice toward reporting ADRs.

**Table 3:** Practice toward ADRs.

Practice of ADRs	Observed ADRs	Report ADRS
At least once a week	24 (14.75)	15 (9.2%)
At least one a month	76 (46.6%)	77 (47.2%)
At least one a year	41 (25.2%)	45 (27.6%)
Never	22 (13.5%)	26 (16%)

The most commonly used method to report ADRs to the SFDA was the online reporting portal (Saudi Vigilance) 92 (56.4%). See Table 4 for more details.

**Table 4:** Methods to report ADRs to SFDA.

Mode of reporting	N (%)
Phone	24 (14.7%)
Fax	9 (5.5%)
Email	38 (23.4%)
SFDA online portal	92 (56.4%)

The respondent's perceptions were then categorized into possible barriers and facilitators to report the ADRs, and the difference between the reporter, non-reporter, observing, and non-observing was identified, as shown in Table 5. The results show that having a reward for reporting ADRs was identified as a key facilitator among community pharmacists who observed previously ADRs in their current practice, *P* = 0.01. In addition, the lack of time was found as a key barrier to reporting ADRs among community pharmacists who previously reported ADRs, *P* = 0.02.

**Table 5:** Facilitators and Barriers to Report ADRs.

	Item	Agree	Difference between reporter and non-reporter *P*-value	Difference between observing and non-observing ADRs *P*-value
Possible facilitators	Having sufficient knowledge and training to report ADRs	82 (50.3%)	0.227	0.516
	Having an incentive/reward for reporting ADRs	88 (54%)	0.914	0.01*
	Making the ADR reporting mandatory for community pharmacists	107 (65.6%)	0.239	0.562
Possible barriers	Lack of time to report ADRs	111 (68.1%)	0.02*	0.228
	Fear of legal consequences from reporting ADRs to SFDA	102 (62.6%)	0.487	0.169
	No actions were taken for the previous ADRs reports sent to SFDA	111 (68.1%)	0.641	0.176

*The value that is less than 0.05 (Significant threshold).

In the final section of the questionnaire, the respondents were asked about what could encourage them to report ADRs to SFDA and improve PV in Saudi Arabia, and several views emerged. The respondents mentioned several suggestions, including conducting of big advertisement campaign about PV via social media for patients, the general public, and healthcare professionals, giving education programs for both patients and community pharmacists, especially new employees, offering incentives to community pharmacists, and updating the information and giving feedback on the previous ADRs reports submitted to SFDA for them to learn. Furthermore, the respondents suggested the implantation of a surveillance system in the big chain pharmacy for patients in order to enable them to collect the ADRs on certain types of medications actively, for example, the new medicines entering the Saudi Drug Market.

## Discussion

This is a cross-sectional assessment of the community pharmacists' knowledge, perception, and current practice toward PV and reporting the ADR to the regulatory health authority in Saudi Arabia (National Pharmacovigilance Center (NPC- SFDA)). Similar to the previous studies, the results of this study have demonstrated that community pharmacists have an adequate level of knowledge regarding PV fields^15,16^. However, there was a clear gap in their knowledge in relation to the health authority responsible for the collection and analysis of ADR reports in Saudi Arabia. Similarly, in 2013, a previous study reported that community pharmacists in Riyadh, Saudi Arabia were unaware of the process of reporting ADRs, including to whom to send reports^17^. In addition, the community pharmacists in the current study demonstrated a gap of knowledge related to the main difference between adverse events and adverse drug reactions and the serious adverse drug reactions that need to be reported immediately to the SFDA. Indeed, community pharmacists were found as the most visited healthcare professionals by the general public^18^; therefore, having an adequate level of knowledge in regard to PV and ADR is essential to assure public safety and quality of the healthcare system in Saudi Arabia.

The community pharmacists in the current study demonstrated positive perceptions of the importance of PV and reporting the ADRs, considering it as a professional obligation, which is similar to the previous studies that assessed the attitude of pharmacists in Saudi Arabia^16^ and other countries, such as the UK and Australia^12,19^. Regarding PV general education, most respondents agreed that PV should be taught in undergraduate pharmacy schools in Saudi Arabia; in fact, several pharmacy schools recently added pharmacoepidemiology courses that encompass PV and medication safety to their new curricula. Previous studies conducted in Pakistan and Malaysia reported that the knowledge and level of awareness toward the importance of ADR reporting were high among pharmacy students compared to students from other medical schools^10,20^. Therefore, including the PV courses in undergraduate pharmacy schools in Saudi Arabia might contribute positively toward the country's future reporting rate of ADRs.

Despite several initiatives and activities (i.e., advertisement campaigns and Post on social media accounts) NPC-SFDA conducted that to highlight the importance of PV and the reporting of ADRs; the respondents in this study also exhibited the need to increase awareness among the general public and community pharmacists. Therefore, additional activities should be considered by the responsible health authority in Saudi Arabia. In order to raise awareness of the importance of PV and reporting ADR, workshops, training sessions, or online continuing education courses in PV could be conducted.

This study also focused on identifying the potential factors that might either hinder or facilitate the reporting of ADR by community pharmacists. Even though most respondents demonstrated a gap in knowledge of the ADR reporting processes to NCP-SFDA, their practice of observing and reporting ADRs was satisfactory. Most respondents in the current study had observed and reported ADRs in their practice (46.6%, and 47.2%, respectively) at least once per month. This finding was contradictory to the previous studies that assessed the ADR reporting practice by community pharmacists in different regions in Saudi Arabia, where a low reporting rate was found^17,21^. This may be due to several factors encompassing the current study, such as a small sample size and targeted community pharmacists across Saudi Arabia. Therefore, the current study's findings warrant careful interpretation, and there is still a need to validate these findings.

Parallel to the previous studies, facilitators and barriers to ADR reporting were considered by community pharmacists, such as lack of time, fear of legal consequences, and lack of feedback on the previous ADR reports. In contrast to the Australian study, the lack of time was a significant barrier among reporting community pharmacists^12^. This could be because the community pharmacists in Saudi Arabia nowadays are responsible for providing several services, for example, assessing the patient's medication management and using the Wasfaty e-prescribing system^22^. However, providing community pharmacists with incentives was found to be a key facilitator among pharmacists who observed but did not report ADRs, and this was opposite to the previous studies that identified providing incentives was mostly highlighted among reporter community pharmacists^12,19^.

In terms of increasing the reporting rate of ADRs, several suggestions were explored, such as simplifying the reporting process. Indeed, community pharmacists in Saudi Arabia are allowed to report ADRs using different ways, either by sending the form online or hardcopy (paper form) and using various communication channels, such as Fax and telephone. However, this seemed not enough for the pharmacists. Therefore, the NPC-SFAD needed to simplify the process further. The NPC-SFDA may take advantage of digital technology and develop a smartphone application to enhance and facilitate the ADRs reporting by healthcare professionals and the public; the use of such an app has resulted in increased reporting rates in several countries like the UK^19^.

This study had a limitation; even though the response rate of this study was low (43%), this was usual for similar national surveys. As well as a self-administered questionnaire being employed, which may result in a recall, honesty, and desirability bias. Therefore, the findings of this study warrant careful interpretation. In addition, this study focused only on the pharmacists working in the community pharmacies; however, a wide range of viewpoints could be obtained from other professionals working in these pharmacies (i.e., managers and owners) to identify potential barriers and facilitators further to enhance the ADRs reporting rate in Saudi Arabia. Finally, this study did not ask about the most seen or reported ADRs, so further study will be needed to investigate this.

## Conclusion

It is evident that the community pharmacist had a positive view regarding the importance of PV and ADR reporting. However, this study identifies a gap in knowledge in a certain aspect of the PV process among working community pharmacists, such as the lack of knowledge of the existing ADRs system and center in Saudi Arabia. Community pharmacists in Saudi Arabia need to be motivated and encouraged to increase the reporting rate of ADRs. Therefore, the NPC-SFDA has to establish further initiatives, such as further education and training programs, proper feedback that needs to be delivered to the reporters, and the use of digital technology innovations to simplify the process and improve the ADR reporting rate.

### Supplementary Information

Supplementary Information.

## Data Availability

Additional data (Pharmacovigilance research tool) is found in the supplementary material. The other data that support the study findings are available upon reasonable request directly from the author: A.K.

## References

[1] Laatikainen O, Sneck S, Bloigu R, Lahtinen M, Lauri T, Turpeinen M (2016). Hospitalizations due to adverse drug events in the elderly-a retrospective register study. Front. Pharmacol..

[2] Mejía G, Saiz-Rodríguez M, Gómez de Olea B, Ochoa D, Abad-Santos F (2020). Urgent hospital admissions caused by adverse drug reactions and medication errors—a population-based study in Spain. Front. Pharmacol..

[3] World Health Organisation . Pharmacovigilance [Internet]. 2002 [cited 2022 Mar 8]. Available from: https://www.who.int/teams/regulation-prequalification/regulation-and-safety/pharmacovigilance

[4] Alshammari TM, Mendi N, Alenzi KA, Alsowaida Y (2019). Pharmacovigilance systems in Arab countries: Overview of 22 Arab countries. Drug Saf..

[5] SFDA. Saudi Vigilance System | Saudi Food and Drug Authority [Internet]. [cited 2022 Mar 8]. Available from: https://www.sfda.gov.sa/en/eservices/65924

[6] Bin Yousef N, Yenugadhati N, Alqahtani N, Alshahrani A, Alshahrani M, Al Jeraisy M (2022). Patterns of adverse drug reactions (ADRs) in Saudi Arabia. Saudi Pharm. J. SPJ.

[7] Aldryhim AY, Alomair A, Alqhtani M, Mahmoud MA, Alshammari TM, Pont LG (2018). Factors that facilitate reporting of adverse drug reactions by pharmacists in Saudi Arabia. Expert Opin. Drug Saf..

[8] AlRuthia, Y., Alsenaidy, M. A., Alrabiah, H. K., AlMuhaisen, A., Alshehri, M. (2018) The status of licensed pharmacy workforce in Saudi Arabia: A 2030 economic vision perspective. Hum. Resour. Health. 16(1). 10.1186/s12960-018-0294-8 PMC6022294 29954408

[9] Alomi YA (2017). New pharmacy model for vision 2030 in Saudi Arabia. J. Pharm. Pract. Commun. Med..

[10] Alqahtani SS, Ahmad S, Alam N, Kashan Syed N, Syed MH, Khardali A (2023). Healthcare professionals' awareness, attitudes and practices towards pharmacovigilance and spontaneous adverse drug reaction reporting in Jazan Province, Saudi Arabia: A survey study. Saudi Pharm. J..

[11] Lancet T (2022). Improving ADR reporting. Lancet.

[12] Li R, Curtain C, Bereznicki L, Zaidi STR (2018). Community pharmacists' knowledge and perspectives of reporting adverse drug reactions in Australia: A cross-sectional survey. Int. J. Clin. Pharm..

[13] Ministray of Health. Statistical Yearbook II-Health Resources [Internet]. 2021 [cited 2023 Aug 16]. Available from: https://www.moh.gov.sa/en/Ministry/Statistics/book/Documents/Statistical-Yearbook-2021.pdf

[14] Sample Size Calculator by Raosoft, Inc. [Internet]. [cited 2023 Aug 16]. Available from: http://www.raosoft.com/samplesize.html

[15] Othman GQ, Ibrahim MIM, Alshakka M, Ansari M, Al-Qadasi F, Halboup AM (2017). Knowledge and perception about pharmacovigilance among pharmacy students of universities in Sana'a Yemen. J. Clin. Diagn. Res..

[16] Alsheikh MY, Alasmari MM (2022). A National survey of community pharmacists' viewpoints about pharmacovigilance and adverse drug reaction reporting in Saudi Arabia. Front. Pharmacol..

[17] Mahmoud MA, Alswaida Y, Alshammari T, Khan TM, Alrasheedy A, Hassali MA (2014). Community pharmacists' knowledge, behaviors and experiences about adverse drug reaction reporting in Saudi Arabia. Saudi Pharm. J. SPJ..

[18] Cheema, E., Haseeb, A., Khan, T. M., Sutcliffe, P., Singer, D. R. (2017) Barriers to reporting of adverse drugs reactions: A cross sectional study among community pharmacists in United Kingdom. Pharm. Pract. (Granada). 15(3). 10.18549/PharmPract.2017.03.931 PMC5597805 28943977

[19] Hughes ML, Weiss M (2019). Adverse drug reaction reporting by community pharmacists—the barriers and facilitators. Pharmacoepidemiol. Drug Saf..

[20] Sman Aamir MU, Mirza AA, Khan S, Anjum M (2018). Knowledge of pharmacovigilance and adverse drug reaction reporting of pharmacy and medical students. Pak. J. Med. Health Sci..

[21] Alghazwani, Y., Alqahtani, A. M., Alshuraymi, M. K., Assiri, I. M., Shuflut, A. A. The perspective of pharmacist on pharmacovigilance and adverse drug reaction reporting in Asir region, Saudi Arabia. 10.26355/eurrev_202302_31410 36876701

[22] Khardali, A., Qadri, M., Alqahtani, S. S. Exploring Community Pharmacists' Attitudes towards the Use of Wasfaty (e-Prescribing) Service in Jazan Province, Saudi Arabia.

