# Supplementary material for: Exploring the knowledge, perception, and practice of community pharmacists in Saudi Arabia toward pharmacovigilance and adverse drug reaction reporting. A nationwide survey
Source: Sci Rep. 2024 Feb 27;14:4819. doi: 10.1038/s41598-024-55664-8 (PMC10899240; doi:10.1038/s41598-024-55664-8)
Supplement: Supplementary file 1 — Supplementary Information. [file 41598_2024_55664_MOESM1_ESM.pdf]

Title: Exploring the Knowledge, Perception, and Practice of Community Pharmacists in Saudi Arabia toward Pharmacovigilance and Adverse Drug Reaction Reporting. A nationwide survey.

Greetings! You are invited to participate in a study being conducted by Dr. Amani Khardali (Assistant Professor, Department of Pharmacy Practice, College of Pharmacy, Jazan University).

The information collected in this survey will be used as part of a research study. There will not be any personally identifiable information collected in this survey, and all responses will be kept in the strictest confidence. Prior to analyzing the data, the order of respondents will be randomized to ensure your responses remain anonymous.

Your participation in this survey is voluntary. You may stop your participation in this survey at any time if you want.

The purpose of this study is to explore the knowledge and opinions of the community pharmacists towards reporting adverse drug reactions in the Saudi Arabia.

If you have any questions or concerns regarding the study, please do not hesitate to contact me at aakherdeli@jazanu.edu.sa

PLEASE ANSWER THE QUESTIONS BELOW BEFORE PROCEEDING TO THE QUESTIONNAIRE. Please indicate by checking the box below that you have read and agree to the above information.

□ I have read and understand the above information and **AGREE** to participate in this research study.

□ I have read and understand the above information and **DO NOT AGREE** to participate in this research study.

I. **Demographic.**

1) Gender □ Male □ Female

2) Age in years ……………………………………………………………………

3) Years of experience ………………………………………………………….

4) Educational Level: □ Pharm.D □ B.Pharm

□ Postgraduate degree in Pharmacy □ Others, Please specify……………………….

II. Knowledge Related to Pharmacovigilance.

1. **What is the definition of pharmacovigilance?**

a. The detection, assessment, understanding, and prevention of adverse reactions
b. The reporting of adverse reactions
c. The science of improving the safety profile of a medicinal product
d. The science of weighing the benefit-risk profile of a medicinal product
e. I do not know.

2. Which of the following is an example of an adverse event?

A. A patient taking Drug X was hospitalized for a stroke. The hospital doctor believes Drug X did not cause the stroke
B. A patient taking Drug X experienced a rash. The rash was believed to have been caused by Drug X
C. A patient taking Drug X said it did not work for him. He did not experience any side effects
D. All of the above
E. I do not know.

3. What type of adverse reactions does the Saudi authority want healthcare professionals to report?

A. All reactions in special populations such as pregnant and breast feeding women, children and elderly.
B. Non-serious adverse reactions.
C. All serious and/or unexpected reactions for well-known drugs.
D. All Adverse reactions that might be related to the use of medicines, vaccines, herbal products, and cosmetics.
E. A, C, and D.
F. I do not know.

4. In Saudi Arabia, which group is currently responsible for the collection and monitoring of ADRs.

A. Drug track and track system (RSD).
B. Drug Establishments National Registry (DENR).
C. The Saudi vigilance system (SVS).

D. Ministry of Health in saudi Arabia.

E. A and C.

F. I do not know.

5. The Saudi Food and Drug authority accepts ADR reports from:

A. Consumers and patients only.

B. Healthcare professionals (physicians, nurses, and pharmacists) only.

C. Hospital networks only.

D. All of the above.

E. I do not know.

6. How do the Saudi Food and drug authority communicate important or new ADR information that has been confirmed?

A. SFDA Early Warning System – monitoring communications.

B. SFDA Medicines Safety Update bulletins.

C. SFDA Risk Management plan.

D. All of the above.

E. I do not know.

7. At which stage is the most adverse event information collected about a drug?

A. Phase I clinical trials.

B. Phase II clinical trials.

C. Phase III clinical trials.

D. Phase IV clinical trials and post-marketing surveillance.

E. I do not know.

8. Please consider the following three statements. Choose the CORRECT answer

A. The terms adverse event and ADR are synonymous and can be used interchangeably.

B. An adverse event is considered as anything medically untoward that happens, and there does not need to be an established causal relationship between the adverse event and the drug.

C. ADRs are collected during clinical trials, whilst adverse events are collected during post-marketing surveillance activities.

I. Only statement A is correct.
II. Only statement B is correct.
III. Only statements A and B are correct.
IV. Only statements B and C are correct.
V. I do not know.

9. Which of the following scenarios would NOT always be considered as a serious adverse event by the SFDA?

A. An adverse event that results in hospitalization.

B. An adverse event that is severe.

C. An adverse event that results in congenital anomaly/birth defect.

D. An adverse event that was considered to be serious by a healthcare professional.

E. I do not know.

10. What is the most common safety reason for withdrawing a drug from the market worldwide?

A. Hepatotoxicity.

B. Cardiotoxicity.

C. Carcinogenicity.

D. Nephrotoxicity.

E. I do not know.

III. Community pharmacists' Perceptions (Likert scale-5).

11. Reporting ADRs is important for patient care

Strongly Disagree Disagree Neutral Agree Strongly Agree

12. Reporting ADRs should be mandatory for community pharmacists.

Strongly Disagree Disagree Neutral Agree Strongly Agree

13. I do not have the time to report ADRs as part of my professional practice.

Strongly Disagree Disagree Neutral Agree Strongly Agree

14. Pharmacovigilance should be taught in the undergraduate pharmacy programs at universities.

Strongly Disagree Disagree Neutral Agree Strongly Agree

15. Professional bodies (e.g., Saudi pharmaceutical society) should organize workshops or training sessions to cover the importance of ADR reporting

Strongly Disagree Disagree Neutral Agree Strongly Agree

16. I currently have sufficient knowledge and training on how to report ADRs.

Strongly Disagree Disagree Neutral Agree Strongly Agree

17. I fear that there may be legal consequences if I report an ADR to the SFDA

Strongly Disagree Disagree Neutral Agree Strongly Agree

18. I have a professional obligation to report ADRs.

Strongly Disagree Disagree Neutral Agree Strongly Agree

19. There are no results or actions taken based on ADRs that I report.

Strongly Disagree Disagree Neutral Agree Strongly Agree

20. I would be encouraged to report more ADRs if it was: Rewarded

Strongly Disagree Disagree Neutral Agree Strongly Agree

21. General education is needed on the importance of pharmacovigilance.

Strongly Disagree Disagree Neutral Agree Strongly Agree

IV. Pharmacist Practice related ADRS.

22. How often do you see ADRs in patients?

a. At least once a week.

b. At least once a month.

c. At least once a year.

d. Never.

23. How often do you record ADRs as part of your clinical interventions?

a. At least once a week.

b. At least once a month.

c. At least once a year.

d. Never.

24. What is the most common method that you use to report an ADR to SFDA?

a. Phone.
b. Fax.
c. Email.
d. SFDA ADRs online reporting portal.
e. I have not reported any ADRs to the SFDA.

25. Finally, do you have any suggestions on what would encourage you to report more ADRs? (free text field).

Thank You
